# Mast cell - tumor cell interaction related gene and microRNA expression profiles in oral squamous cell carcinoma

**DOI:** 10.3389/fonc.2025.1518404

**Published:** 2025-02-21

**Authors:** Tatjana Khromov, Maren Sitte, Gabriela Salinas, Boris Schminke, Andreas Fischer, Henning Schliephake, Phillipp Brockmeyer

**Affiliations:** ^1^ Department of Clinical Chemistry, University Medical Center Goettingen, Goettingen, Germany; ^2^ Department of Oral and Maxillofacial Surgery, University Medical Center Goettingen, Goettingen, Germany; ^3^ NGS- Integrative Genomics Core Unit, Institute of Pathology, University Medical Center Goettingen, Goettingen, Germany

**Keywords:** oral squamous cell carcinoma, OSCC, mast cells, tumor microenvironment, prognosis, CC chemokine ligand 2, CCL2, CCR2

## Abstract

**Backround:**

Mast cells (MCs) play a crucial role in the tumor microenvironment (TME) of oral squamous cell carcinoma (OSCC), significantly impacting patient prognosis. This study aimed to investigate the gene and microRNA (miRNA) expression profiles of MCs and OSCC cells following co-culture, providing valuable insights into the molecular background of their functional interactions.

**Methods:**

The human OSCC cell line PCI-13 and the human MC cell line LUVA were initially cultured separately under identical experimental conditions and subsequently co-cultured for 48-72h. Transcriptome analysis of differentially expressed genes (DEGs) and sequencing of differentially expressed miRNAs were performed and analyzed using bioinformatics tools. Additionally, key genes and miRNAs identified in OSCC were assessed for their prognostic relevance in head and neck tumors using freely available online databases.

**Results:**

The analyses revealed distinct DEG profiles between OSCC cells and MCs under monoculture and co-culture conditions. Notable findings include DEGs involved in chemokine signaling - particularly the CCL2/CCR2 axis - TGF-β signaling, toll-like receptor (TLR) expression, and key intracellular pathways such as PI3K/Akt, JAK/STAT, Ras/Raf/MAPK, and IP3 in both cell types. Additionally, specific miRNAs, including miR-142, miR-146a, and miR-223 in tumor cells, as well as miR-381 and miR-379 in MCs, exhibited significant differential expression, highlighting their potential involvement in regulating MC-OSCC interaction. Notably, the expression levels of CCR2, along with miR-142, miR-146a, and miR-223, were identified as prognostically relevant in patients suffering from head and neck tumor.

**Conclusions:**

The data highlight the complex and dynamic interplay between MCs and OSCC, driven by key signaling pathways and miRNA regulation. These findings provide a foundation for future functional studies and the possible development of targeted therapies aimed at modulating MC-OSCC interaction within the TME.

## 1 Introduction

Oral squamous cell carcinoma (OSCC) represents a significant proportion of malignant head and neck tumors (1), a heterogeneous group of diseases that are among the most common tumors worldwide (2). Patients with advanced disease have a poor prognosis (1), with a high probability of loss of function (chewing, swallowing, speaking) (3), poor facial aesthetics, and a corresponding reduction in health-related quality of life (4). One of the main reasons for the unfavorable prognosis is invasion and metastasis (5), which is influenced by tumor cell-specific changes associated with an epithelial-mesenchymal transition (EMT) process and the tumor microenvironment (TME) (6). The TME is composed of a variety of cells, including immune cells such as macrophages, lymphocytes, and granulocytes (7). The interaction of OSCC with the immune system is currently being investigated (8), and immunotherapeutic drugs have been approved for the treatment of OSCC (9), but not all patients benefit, highlighting the need for further identification of alternative therapeutic approaches (10).

Mast cells (MCs) are key components of the immune system, located primarily under the skin and mucous membranes (11, 12). They develop from pluripotent progenitor cells in the bone marrow and mature under the influence of c-Kit ligand and stem cell factor (SCF) (13), and also various growth factors provided by the microenvironment (14). MCs are known to play a critical role in immediate hypersensitivity reactions (15), bacterial, viral, parasitic and fungal infections, as well as autoimmune diseases (16).

In the oral mucosa, they contribute to immune responses and inflammation by releasing mediators such as histamine, cytokines, and proteases, thereby influencing both innate and adaptive immunity (17–20). MC density correlates with disease severity and inflammation in conditions such as oral lichen planus and OSCC (21–23). MC density has been moreover shown to be associated with risk factors such as smoking and alcohol consumption (24, 25). They recruit different types of immune cells, modulate tissue repair, and release angiogenic factors that promote neovascularization in pathological conditions (19, 21, 26, 27). Thus, MCs are essential for oral mucosa homeostasis and the pathogenesis of oral diseases (18, 22, 28).

In addition, MCs have a significant impact on tumor biology with tumor-promoting and tumor-inhibiting effects, and an influence on patient prognosis has been observed in various tumor entities (29–38). These different functions appear to depend on the localization of MCs, whether they are able to infiltrate the inner tumor mass or are more distantly located in the TME (30, 35, 36, 38–46).

MCs have the ability to infiltrate a variety of solid tumors, where they are termed tumor-associated MCs (TAMCs) (47). Subsequently, they are found in close proximity to tumor cells, where they facilitate a variety of processes that contribute to tumor progression (47). They synthesize and store angiogenic factors and matrix metalloproteinases that promote tumor vascularization or invasiveness (47). In addition, histamine release can induce tumor cell proliferation (48). A reduction in the number of intratumorally localized TAMCs has been shown to correlate with improved antitumor response and patient prognosis (49). However, MCs located at a greater distance from tumor cells in the TME, are able to translate stimuli from the local environment into the regulated secretion of active chemical mediators by activating specific signaling pathways associated with different secretion pathways (50). This has a direct impact on the cellular characteristics of the tumor, including proliferation, migration, and invasion (50). MCs can inhibit cancer growth, promote cellular apoptosis, and suppress type 1 T helper cell immune responses by releasing cytokines such as IL-1, -4, and -6, transforming growth factor β (TGF-β), and tumor necrosis factor α (51). MC tryptases can induce tumor cell destruction and inflammation by activating protease-activated receptor 2 (52). In addition, cells of the TME, including MCs and tumor cells, communicate and influence each other via microRNA (miRNA)-containing extracellular vesicle (EVs) transfer to mutually modulate the expression of specific genes and influence intracellular signaling (50, 53, 54). In addition, EV release by MCs can attract additional immune cells, including T and B lymphocytes, to the tumor region to exert anti-tumor functions (55–57).

In an initial pilot study analyzing tissue samples from 118 patients, immunohistochemistry was conducted using MC tryptase and CD117 markers (58). We identified a significantly higher MC density within the TME of OSCC in patients with better prognoses (58). Additionally, our *in vitro* experiments demonstrated that MCs influence the proliferative and invasive characteristics of OSCC, with the cytokine CC chemokine ligand 2 (CCL2) identified as a potential mediator (59). CCL2 is a potent chemoattractant for immune cells, including macrophages, myeloid-derived suppressor cells (MDSC), mesenchymal stem cells (MSC), and regulatory T cells (Treg), via the corresponding CCR2 receptor on the cell surface (60). It also serves as a potent inducer of chronic inflammation within the TME (60) and is associated with intracellular signaling that can alter tumor cell characteristics such as proliferation, migration and chemotaxis (61).

To gain a more detailed molecular understanding of the prognosis-relevant interplay between MCs and OSCC and to uncover targets for potential therapeutic intervention, it is crucial to analyze the changes in gene and miRNA expression profiles of both cell types. In this study, we identified several differentially expressed genes (DEGs) and miRNAs that influence intracellular signaling and may drive the MC-OSCC crosstalk.

## 2 Materials and methods

### 2.1 Cell culture

**Figure 1:**
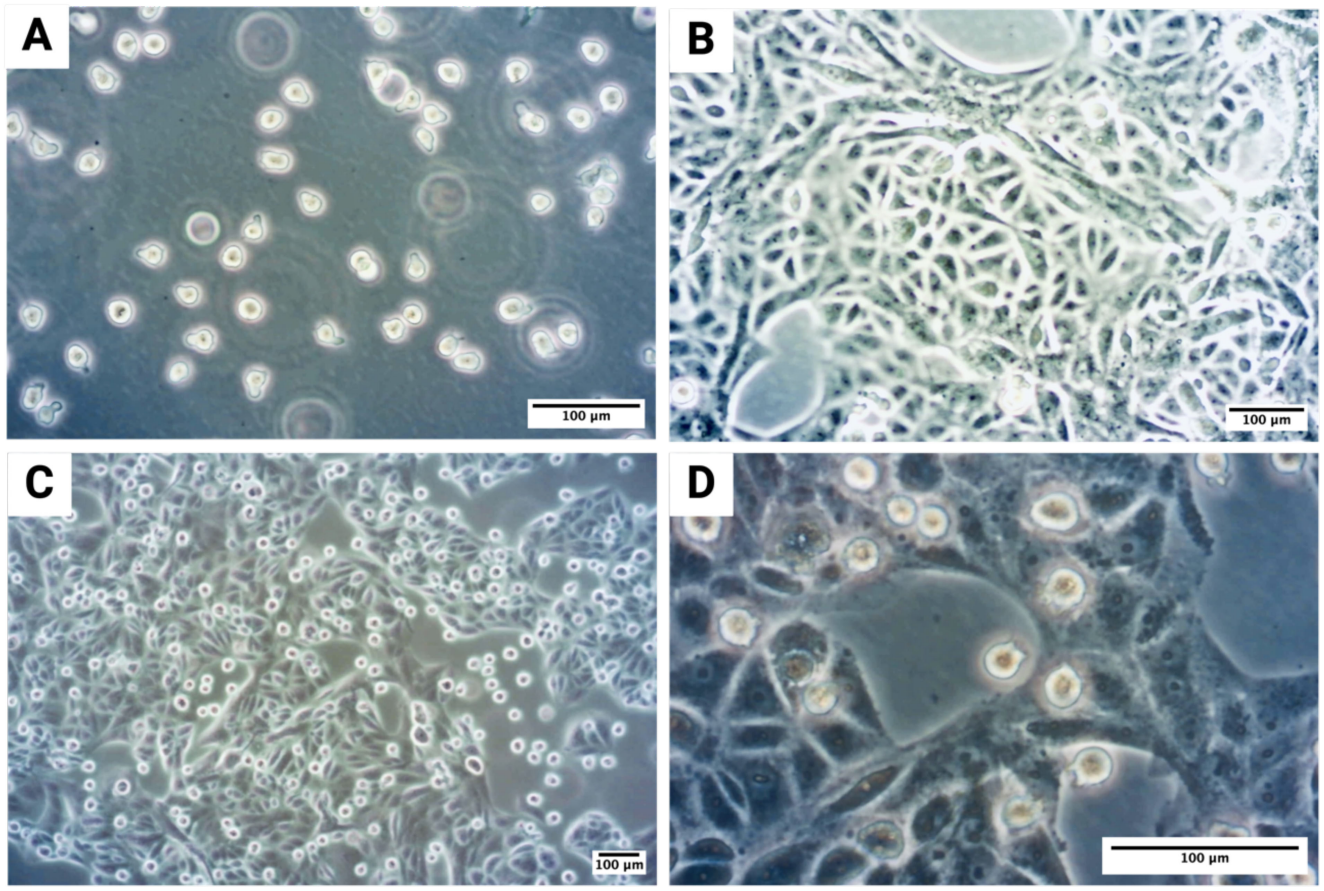
Microscopic visualization of MC-OSCC co-culture experiments: **(A)** Monoculture of the human MC cell line LUVA, a non-adherent suspension cell line. **(B)** Monoculture of the OSCC tumor cell line PCI-13, adapted to stem cell medium to ensure comparable experimental conditions, showing distinct tumor cell clusters. **(C)** Overview of the co-culture of PCI-13 tumor cells and LUVA MCs in stem cell medium for 48–72h. **(D)** Higher magnification of the co-culture experiment.

The human OSCC cell line PCI-13 was obtained from the University of Pittsburgh Cancer Institute (UPCI, Pittsburgh, PA, USA) (62), and the CD34+, c-kit+, and CD13+ human MC cell line LUVA was obtained from Kerafast (Boston, MA, USA). LUVA cells were cultured with StemPro™-34 SFM medium enrichted with StemPro™-34 Nutrient Supplement (Thermo Fisher Scientific), 200 mM L-glutamine (Sigma Aldrich, St. Louis, MO, USA), and 1% penicillin-streptomycin (PAN Biotech, Aidenbach, Germany) (**Figure 1A**). PCI-13 cells were initially cultured in Dulbecco’s modified Eagle’s medium (DMEM) supplemented with GlutaMAX (Thermo Fisher Scientific, Waltham, MA, USA), 10% heat-inactivated fetal bovine serum (FBS) (Biochrom, Berlin, Germany), and 1% penicillin-streptomycin (PAN Biotech, Aidenbach, Germany). Later, PCI-13 cells were transferred to supplemented StemPro™-34 SFM to ensure comparable experimental conditions (**Figure 1B**). Both cell lines were grown in T175 flasks at 37°C in a humidified incubator containing 5% CO_2_.

### 2.2 Mast cell – tumor cell co-cultures

For co-culture experiments, the OSCC cell line PCI-13 (1 × 10^6^ cells per flask) was cultured in StemPro™-34 SFM medium, allowing the cells to adhere to the bottom of the flask. After 24h, the medium was changed, and the LUVA cell line (1 × 10^6^ cells per flask) was added to the same flask for co-culture. The co-culture was maintained for 48-72h as shown in **Figure 1C**. PCI-13 cells formed a monolayer at the bottom of the flask, while LUVA cells remained suspended in the medium (**Figure 1D**). To separate the cell lines, the medium containing the LUVA cells was collected and centrifuged at 200×g for 10 minutes at room temperature. The PCI-13 cell monolayer was detached by trypsinization, and the resulting cell pellets were collected. Both media and cell pellets were aliquoted and stored at -80°C for subsequent analysis.

### 2.3 RNA isolation, RNA-Seq and miRNA library preparation

RNA isolation was performed using Trizol reagent (Thermo Fisher Scientific, Waltham, MA, USA) according to the manufacturer’s instructions. RNA quality was assessed by measuring the RNA integrity number (RIN) using a Fragment Analyzer HS Total RNA Kit (Advanced Analytical Technologies, Inc.). Library preparation for RNA-Seq was performed on the STAR Hamilton NGS automation system using the Illumina Stranded Total RNA Prep, Ligation with Ribo-Zero Plus (Cat. No. 20040525) and the ID for Illumina RNA UD Indexes Set A, Ligation with 96 Indexes (Cat. No. 20040553) with an input of 300 ng total RNA. The size range of the final cDNA libraries was determined using the SS NGS Fragment 1 to 6000 bp Kit on the Fragment Analyzer, with an average size of 340 bp. The cDNA libraries were accurately quantified using the DeNovix DS series systems. The construction of miRNA libraries was performed using the QIAseq miRNA Library Kit (Cat. 331505 Qiagen) with a total starting amount of RNA of 500 ng. The kit incorporates a number of measures to mitigate potential biases inherent in the reaction process. These include the avoidance of cutting gel techniques for miRNA se-lection and the elimination of adapter dimer formation. In addition, unique molecular indices (UMIs) were assigned to each miRNA at an early stage, eliminating potential biases associated with polymerase chain reaction (PCR) and sequencing.

### 2.4 mRNA and miRNA sequencing

The final cDNA and miRNA libraries were sequenced using an S2 flow cell on a NovaSeq 6000 instrument (Illumina, San Diego, CA, USA, mRNA: 100 cycles, 25 million reads/sample, miRNA: 100 cycles (2x50 bp), 5-8 million reads/sample). Sequence images were converted to BCL files using BaseCaller software (Illumina, San Diego, CA, USA), which were demultiplexed to fastq files using bcl2fastq v2.20 (Illumina, San Diego, CA, USA). Sequence quality was checked using FastQC software (v. 0.11.5; **http://www.bioinformatics.babraham.ac.uk/projects/fastqc/** ) (63).

### 2.5 RT-qPCR validation

To validate key and highly differentially expressed genes (DEGs) identified from the mRNA sequencing analysis (CCL2, CCR2, SKIL, MLC1, MT-ND2 and MT-CYB), RT-qPCR was performed. RNA was extracted from samples using the Trizol reagent (Thermo Fisher Scientific, Waltham, MA, USA). RNA concentration and quality were assessed using a DS-11 FX+ spectrophotometer/fluorometer (DeNovix, Wilmington, DE, USA). cDNA was synthesized from 1 µg of total RNA using the iScript cDNA synthesis kit (Bio-Rad, Hercules, CA, USA). Gene-specific primers for RT-qPCR were designed using Primer3web software (version 4.1.0, primer3.ut.ee, **Table 1**). Primer specificity was confirmed via melting curve analysis. RT-qPCR was carried out using the LightCycler 480 II (Roche, Heidelberg, Germany) with PowerUp SYBR Green Master Mix (applied biosystems, Foster City, CA, USA) under the following cycling conditions: initial denaturation step at 95°C for 3 min, followed by 40 amplification cycles at 95°C for 20 s, annealing at 58°C for 15 s, extension at 72°C for 10 s and final elongation at 81°C for 5 s. After PCR, a postamplification melting curve program was initiated by heating to 95°C for 20 s, cooling to 65°C for 10 s and increasing the temperature to 95°C continuously. Each PCR run contained a negative (no template) control and each amplicon done in triplicate. Relative gene expression levels were determined using the comparative ΔΔCt method, with monocultured cells serving as calibrators in each experiment. The relative quantity (RQ) of target gene expression between samples was normalized to the housekeeping gene GAPDH, which was used as an internal control. All experiments were conducted in triplicate to ensure reproducibility. Statistical significance was evaluated using student’s t-test.

**Table 1:** Primer sequences.

Gene	Sense-Sequence	Anti-Sense Sequence
CCL2 CCR2 SKIL MLC1 MT-ND2 MT-CYB GAPDH	GAGGCTGAGACTAACCCAGA ACGGTGCTCCCTGTCATAAA AGCAGGAAGGTGACCATGTT GAGCCATTCAGAGAGGAGCT CCCAGCCTACTCCTCAATCA TGAAACTTCGGCTCACTCCT GAGTCAACGGATI TGGTCGT	GGTGACTGGGGCATTGATTG TCAGAGATGGCCAGGTTGAG AGCCTTCTCTGACTGTCTTGA TGAAGCTCACAATTGCCGAG AGGCATGAGATAGTGACAGGG CCGATGTGTAGGAAGAGGCA GACAAGCTTCCCGTTCTCAG

### 2.6 Statistics

#### 2.6.1 Differentially expressed genes

Sequences were aligned to the Homo sapiens reference genome (GRCh38.p13, https://www.ensembl.org/Homo_sapiens/Info/Index) using the RNA-Seq alignment tool STAR version 2.7.8 (64), allowing for 2 mismatches. Read counts were then performed using Feature-Counts (65). Read counts were analyzed in the R/Bioconductor environment (version 4.4.0, www.bioconductor.org) using the DESeq2 package version 1.44.0 (66). Candidate genes were filtered using absolute log_2_ fold change >1 and FDR-corrected p-value <0.05. Potentially regulated genes were selected for gene set enrichment analysis (GSEA) embedded in the R package WebGestaltR (67). For the individual differential expression results, a rank score was calculated for each gene. These ranks were then passed to the GSEA as implemented in the R package WebGestaltR (67). Key genes in OSCC were evaluated for already known influence on overall survival (OS) in head and neck tumors using the freely available online GEPIA 2 platform (68).

#### 2.6.2 Differentially expressed miRNAs

Sequences were aligned to the Homo sapiens reference genome (GRCh38.p13, https://www.ensembl.org/Homo_sapiens/Info/Index) using Bowtie2 version 2.3.4 (69) and Samtools version 1.9 (70). Expression quantification was performed using Salmon in quant mode (71). Read counts were analyzed in the R/Bioconductor environment (version 4.4.0, www.bioconductor.org) using the DESeq2 package version 1.44.0 (66). Candidate miRNAs were filtered by absolute log_2_ fold change >1 and FDR-corrected p-value <0.05. To investigate KEGG pathways regulated by miRNA candidates, we performed pathway analysis using the miRPathDB 2.0 online tool (72). The following search parameters were selected: evidence - experimental (weak+strong); minimum number of significant pathways a miRNA should show - 1; and minimum number of significant miRNAs a pathway should show - 2. Key miRNAs in OSCC were evaluated for their known influence on OS in head and neck tumors using the freely available online OncomiR platform (73).

## 3 Results

### 3.1 Key gene signatures in MC-OSCC interaction

**Figure 2:**
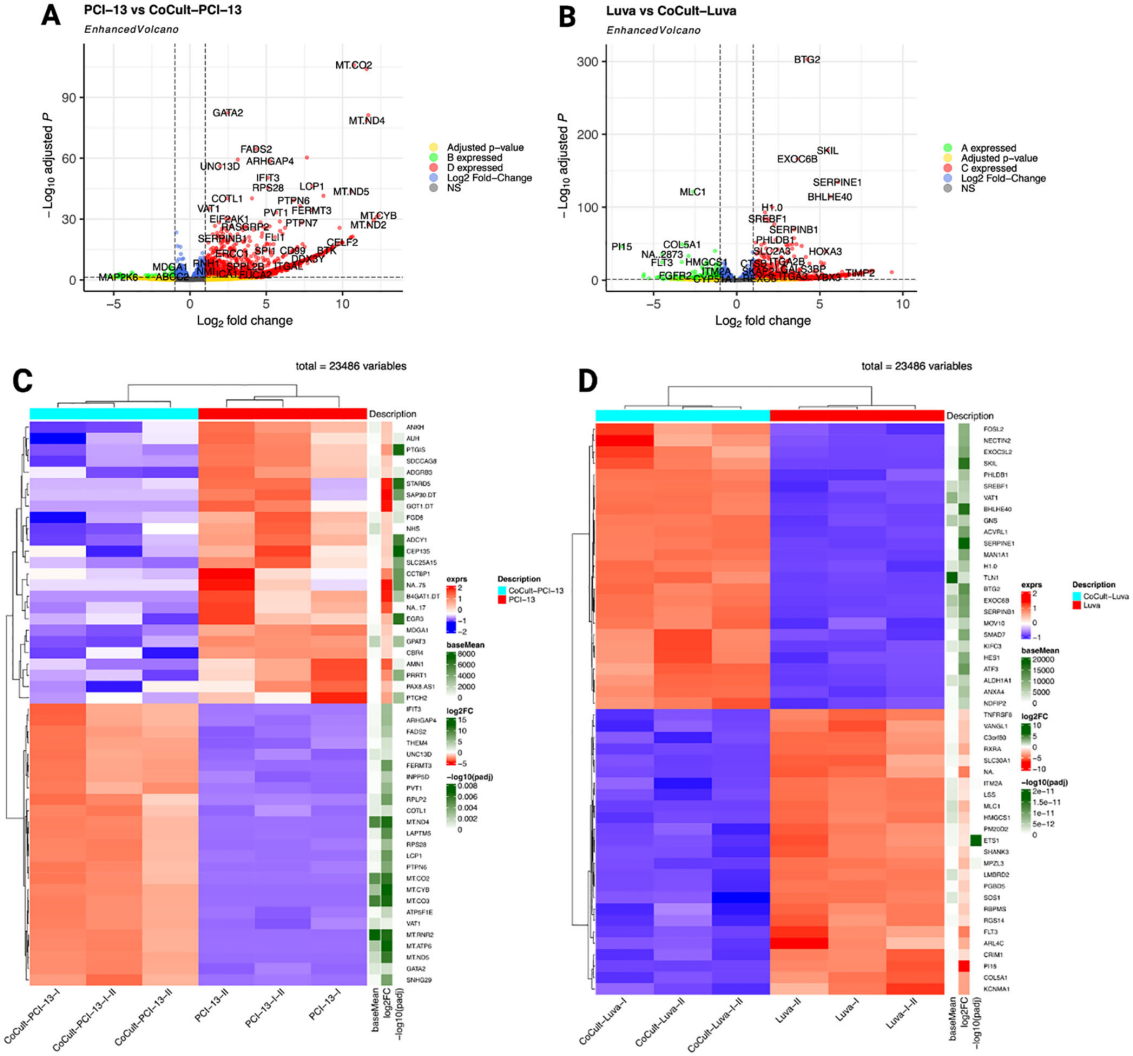
Differential gene expression analysis comparing monocultured OSCC cells (PCI-13) and MCs (LUVA) with their respective counterparts after 48-72h of co-cultivation. **(A)** Enhanced volcano plot highlighting DEGs in OSCC cells. The x-axis represents the log_2_ fold change in expression and the y-axis shows the -log_10_ adjusted p-value. Each point represents a single gene. Genes with larger absolute log_2_ fold changes and higher -log_1_ adjusted p-values are considered to be more significantly differentially expressed. Notably, MT-CO2, MT-ND2, MT-ND4, MT-ND5, and MT-CYB exhibit significant overexpression, while MAP2K6, ABCC2, and MDGA demonstrate pronounced downregulation. **(B)** DEG analysis in MCs, revealing significant overexpression of BTG2, SKIL, EXOC6B, SERPINE1, and BHLHE40, alongside marked downregulation of MLC1. **(C)** Heatmap depicting the 50 most deregulated genes in OSCC cells (PCI-13-I, PCI-13-II, and PCI-13-I-II) compared to co-cultured OSCC cells (CoCult-PCI-13-I, CoCult-PCI-13-II, CoCult-PCI-13-I-II). Gene expression levels are color-coded, with blue representing downregulation and red representing overexpression. Hierarchical clustering reveals distinct transcriptional profiles driven by the MC-OSCC interplay, underscoring both shared and unique transcriptomic changes among the three OSCC variants. **(D)** Heatmap displaying the 50 most deregulated genes in MCs under monoculture conditions (Luva-I, Luva-II, Luva-I-II) compared to co-cultured MCs (CoCult-Luva-I, CoCult-Luva-II, CoCult-Luva-I-II). These findings highlight significant transcriptional alterations induced by MC-OSCC interaction, suggesting changes in distinct biological mechanisms and cellular crosstalk underlying the observed gene expression changes.

A total of 23,486 genes were analyzed using a fold-change threshold of 1 and an adjusted p-value cut-off of 0.05. Of these, 7.31% were upregulated and 0.31% were downregulated in OSCC cells. In contrast, 4.27% of genes were upregulated and 0.92% were downregulated in MCs. The volcano plots (**Figures 2A, B**) illustrate the global distribution of DEGs, highlighting key candidates based on Log_2_ fold-change and statistical significance (-Log_10_ adjusted p-value). OSCC cells exhibited significant upregulation of mitochondrial genes, including MT-CO2, MT-ND2, MT-ND4, MT-ND5, and MT-CYB, suggesting metabolic adaptation (74, 75). Conversely, the significant downregulation of MAP2K6, ABCC2, and MDGA suggests impaired MAPK signaling, which may affect tumor cell proliferation, differentiation, and drug resistance (76, 77). In MCs, notable upregulated genes included BTG2, SKIL, EXOC6B, SERPINE1, and BHLHE40, suggesting that co-culture with OSCC cells alters transcriptional programs related to immune response and cellular differentiation (78–80). Meanwhile, the downregulation of MLC1 suggests a possible regulation of the immune response (81). Heatmaps of the top 50 deregulated genes (**Figures 2C, D**) show distinct transcriptional clustering, clearly delineating transcriptional differences between monoculture and co-culture conditions.

### 3.2 RT-qPCR validation confirms transcriptomic findings

**Figure 3:**
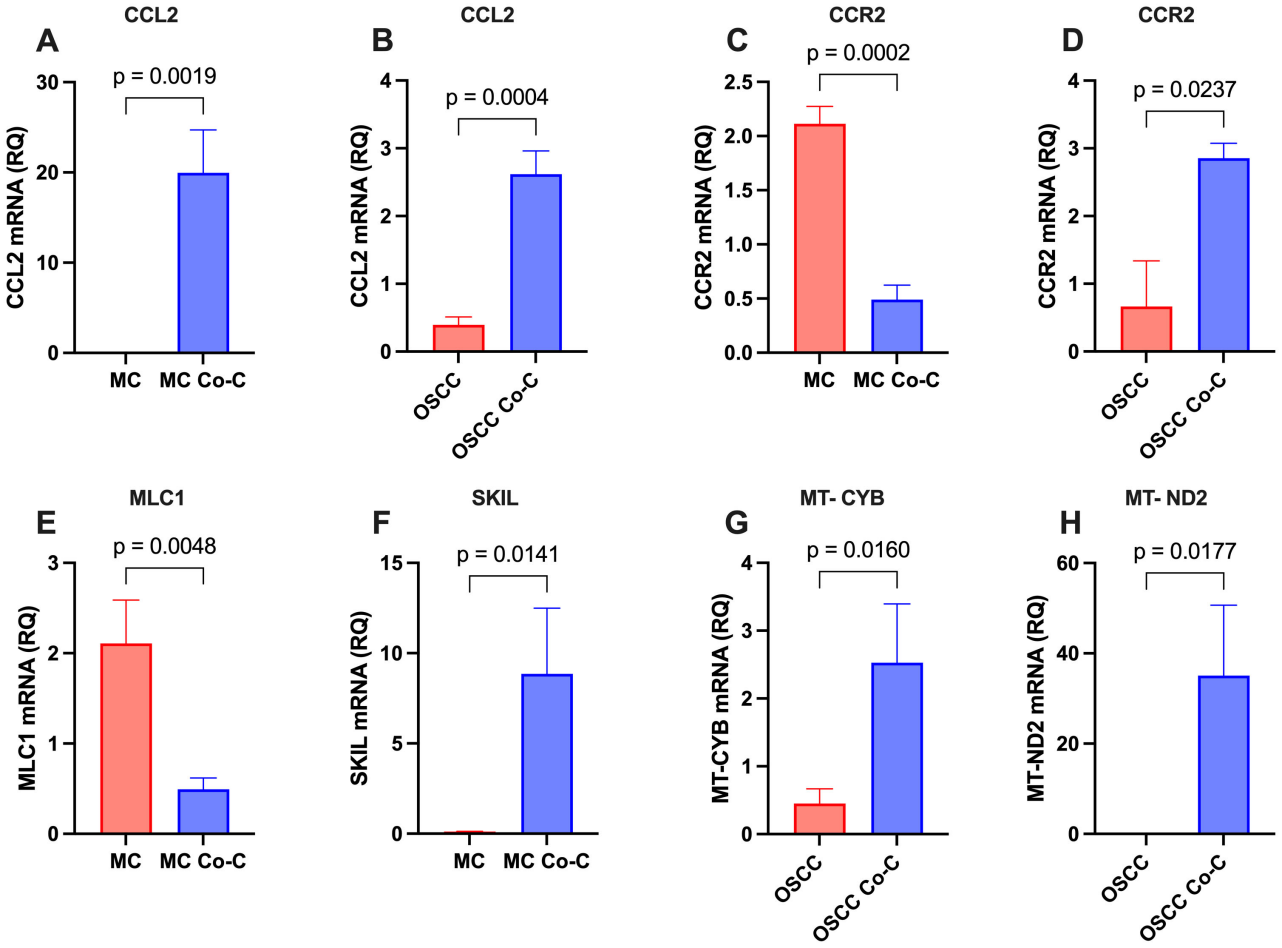
Validation of key and highly differentially expressed genes in monocultured (MC and OSCC) and co-cultured (MC Co-C and OSCC Co-C) cells. Cells were cultured either individually in stem cell medium or co-cultured for 48-72h. Total RNA was extracted and analyzed by RT-qPCR using gene-specific primers (**Table 1**, n = 3 per condition). The relative quantity (RQ) of gene expression is presented for: **(A)** CCL2 in MCs, **(B)** CCL2 in OSCC cells, **(C)** CCR2 in MCs, **(D)** CCR2 in OSCC cells, **(E)** MLC1 in MCs, **(F)** SKIL in MCs, **(G)** MT-CYB in OSCC cells, and **(H)** MT-ND2 in OSCC cells. Statistical significance was assessed using Student’s t-test.

To validate the transcriptomic findings, RT-qPCR was conducted on a subset of key and highly deregulated genes (**Figure 3**). In MCs, the analysis confirmed the upregulation of CCL2 and SKIL, as well as the downregulation of CCR2 and MLC1. In OSCC cells, upregulated genes included CCL2, CCR2, MT-CYB, and MT-ND2. The RT-qPCR expression levels showed a strong correlation with the RNA-seq data, further validating the reliability of the transcriptomic analysis.

### 3.3 Key signaling pathways and biological processes involved in MC-OSCC interactions

**Figure 4:**
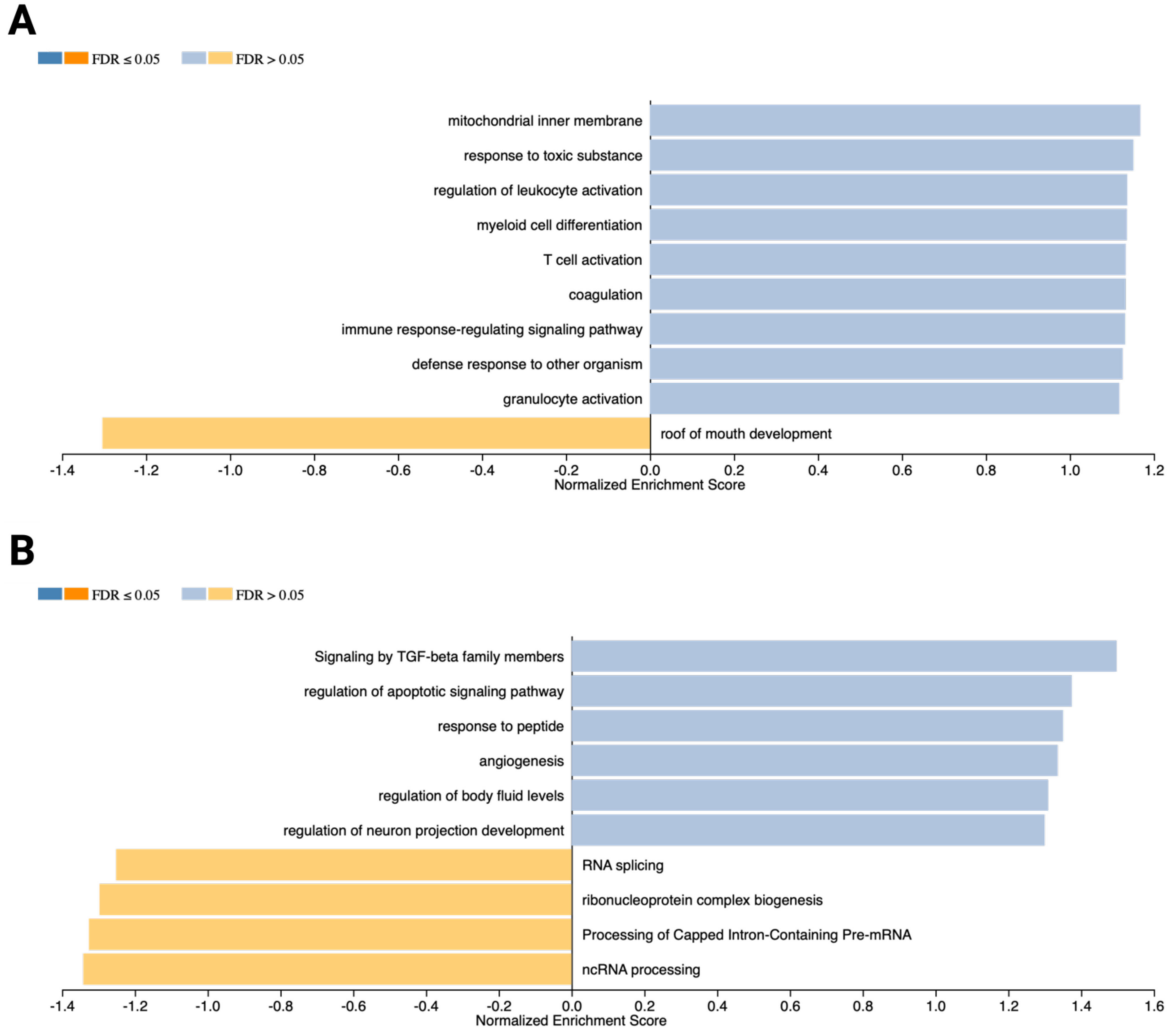
Gene Set Enrichment Analysis (GSEA) results generated using the WebGestaltR R package. **(A)** Bar chart illustrating weighted gene clusters corresponding to enriched pathways and biological processes in OSCC cells (PCI-13). Normalized enrichment scores (NES) and FDR-adjusted p-values are shown. Overrepresented pathways are highlighted in blue, while underrepresented pathways are depicted in orange. **(B)** GSEA results for MCs (LUVA), showcasing pathways and biological processes with significant enrichment.

Gene Set Enrichment Analysis (GSEA) identified pathways and biological processes significantly enriched among the DEGs. In OSCC cells, the enrichment plot (**Figure 4A**) highlights pathways associated with mitochondrial inner membrane regulation, response to toxic substances, leukocyte activation, myeloid cell differentiation, T cell activation, coagulation, immune signaling, defense responses to other organisms, and granulocyte activation (FDR > 0.05). In contrast, the roof of mouth development pathway was downregulated (FDR > 0.05). For MCs, the enrichment plot (**Figure 4B**) shows overrepresentation of pathways related to TGF-β family signaling, regulation of apoptotic signaling, peptide response, angiogenesis, regulation of body fluid levels, and neuron projection development (FDR > 0.05). Conversely, processes such as RNA splicing, ribonucleoprotein complex biogenesis, capped intron-containing pre-mRNA processing, and ncRNA processing were downregulated (FDR > 0.05).

### 3.4 Key miRNAs regulating MC-OSCC interactions

**Figure 5:**
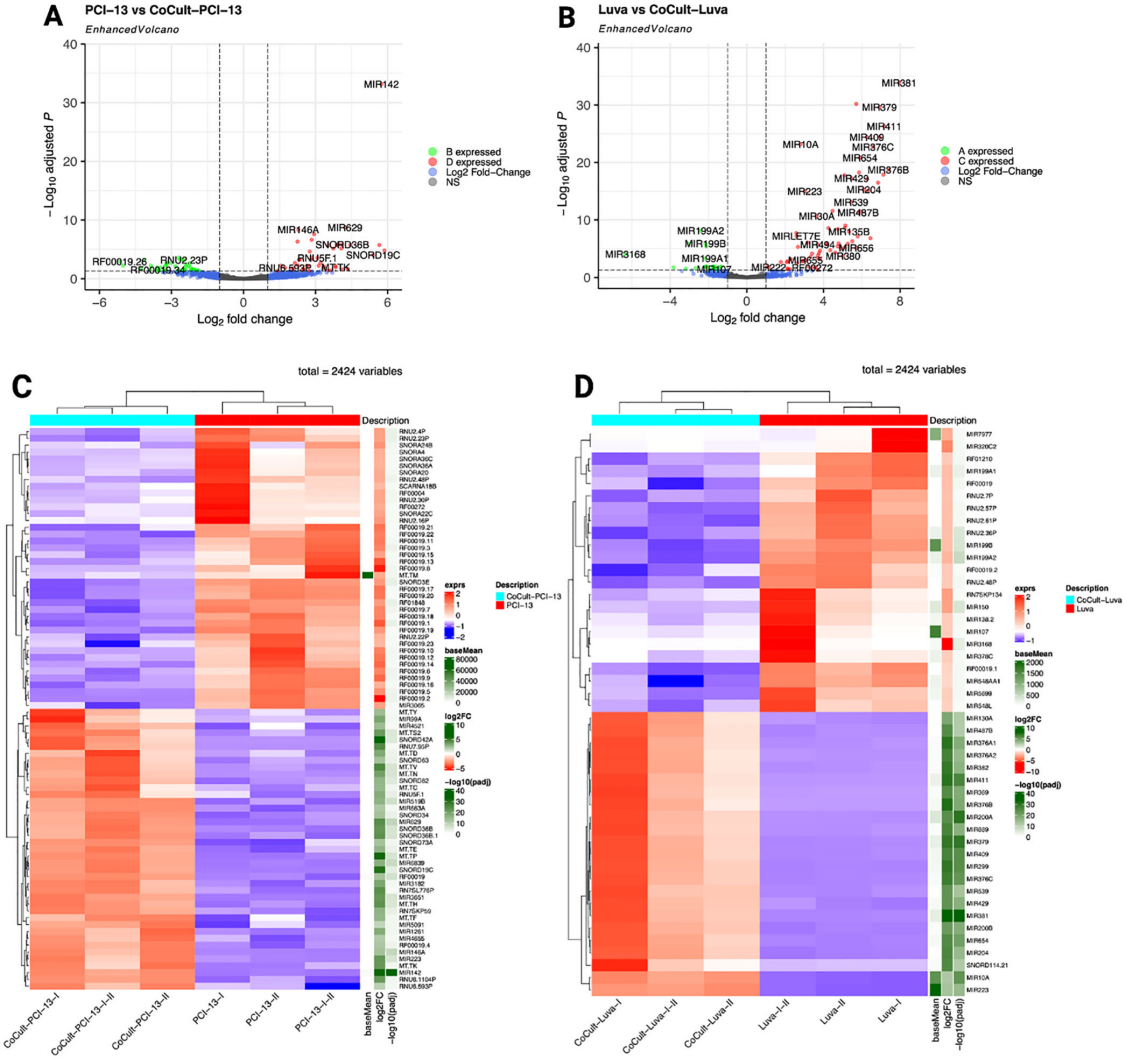
miRNA sequencing analysis comparing monocultured MCs (LUVA) and OSCC cells (PCI-13) with their respective counterparts after 48-72h of co-cultivation. **(A)** Enhanced volcano plot displaying differentially expressed miRNAs in OSCC cells. The x-axis represents the log_2_ fold change in expression and the y-axis shows the -log_10_ adjusted p-value. Each point represents one miRNA. miRNAs with larger absolute log_2_ fold changes and higher -log_1_ adjusted p-values are considered to be more significantly differentially expressed. Notable candidates include miR-142, miR-629, and miR-146a, which exhibit the most significant overexpression. **(B)** Enhanced volcano plot for MCs showing miR-381, miR-379, and miR-411 as the most significantly overexpressed miRNAs, while miR-3168 displays the highest downregulation. **(C)** Heatmap illustrating the top 50 deregulated miRNAs in monocultured OSCC cells (PCI-13-I, PCI-13-II, PCI-13-I-II) compared to co-cultured OSCC cells (CoCult-PCI-13-I, CoCult-PCI-13-II, CoCult-PCI-13-I-II). miRNA expression is represented by a color gradient, with blue indicating downregulation and red indicating overexpression. Key deregulated miRNAs, such as miR-223, miR-146a, and miR-142, suggest potential mechanisms by which MCs modulate the OSCC cell transcriptome. **(D)** Heatmap depicting the top 50 deregulated miRNAs in monocultured MCs (Luva-I, Luva-II, Luva-I-II) compared to co-cultured MCs (CoCult-Luva-I, CoCult-Luva-II, CoCult-Luva-I-II). This analysis highlights significant expression shifts in key miRNAs, including miR-223, miR-199a1, and miR-150, indicating their potential involvement in the MC-OSCC interaction and regulation of cellular processes.

miRNA sequencing revealed a distinct set of deregulated miRNAs under MC-OSCC co-culture conditions compared to monocultured MCs and OSCC cells, highlighting their regulatory roles in MC-OSCC interactions. A total of 2,424 miRNAs were analyzed using a fold-change threshold of 1 and an adjusted p-value cutoff of 0.05. This analysis identified 1.86% of miRNAs as upregulated and 1.69% as downregulated in OSCC cells. In MCs, 2.97% of miRNAs were upregulated, while 0.95% were downregulated. The volcano plot (**Figure 5A**) highlights miR-142, miR-146a, and miR-629 as key candidates in OSCC cells. These miRNAs are known for their tumor-suppressor or oncogenic roles and their involvement in processes such as immune regulation, cell proliferation, apoptosis, and metastasis (82–84). In MCs, the volcano plot (**Figure 5B**) identifies miR-381 and miR-379 as the most significantly overexpressed candidates, while miR-3168 emerges as the most prominently downregulated miRNA. These miRNAs are implicated in post-transcriptional regulation and immune cell modulation, further suggesting their contributions to the observed transcriptomic changes (85–87). The heatmaps of the top 50 deregulated miRNAs in OSCC cells and MCs (**Figures 5C, D**) reveal distinct clustering patterns, underscoring their differential expression profiles and potential co-regulatory functions. These findings underscore the critical role of miRNA-mediated regulation in modulating MC-OSCC interactions. They complement the transcriptomic changes observed at the mRNA level and provide a foundation for future mechanistic investigations.

### 3.5 Regulatory miRNAs modulate key pathways in MC-OSCC interactions

**Figure 6:**
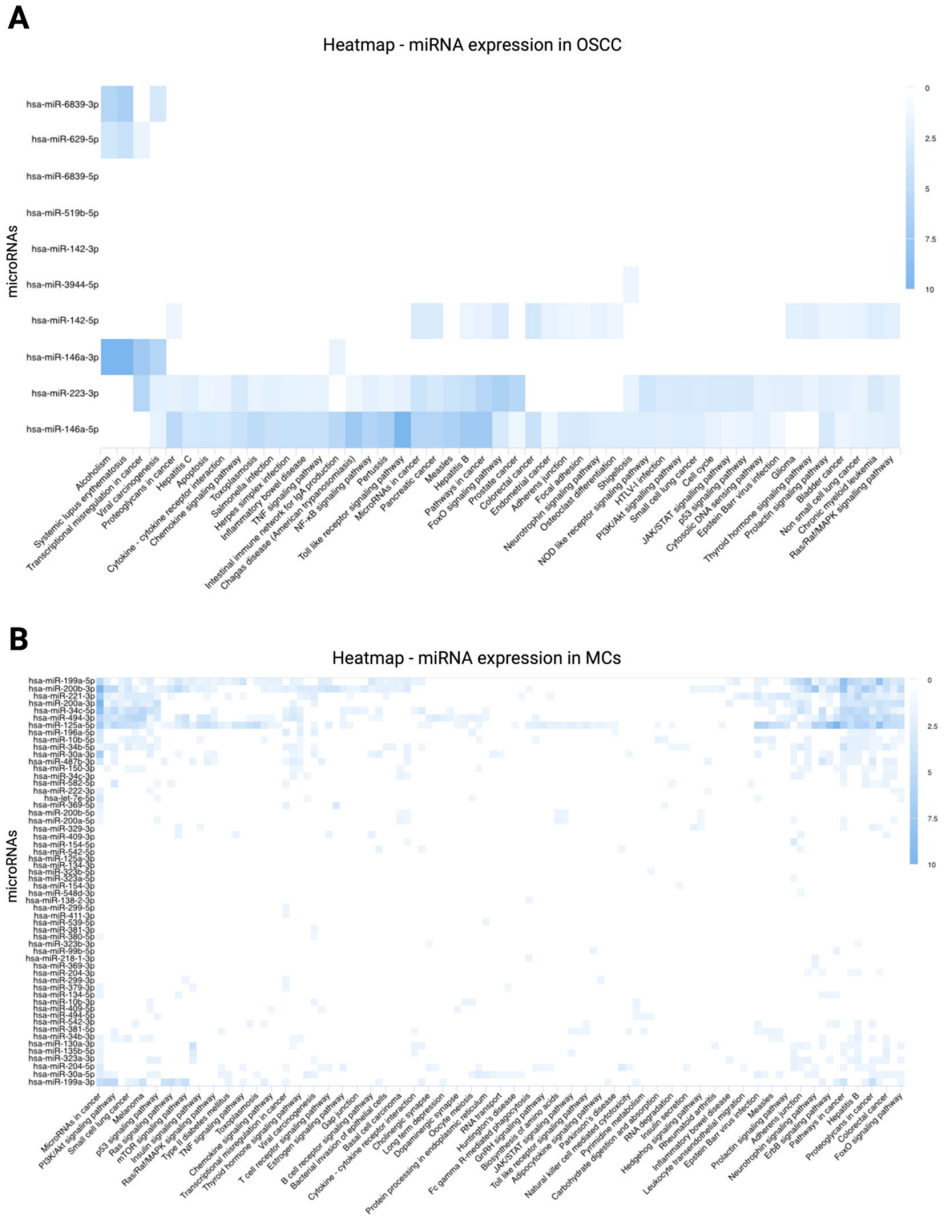
KEGG pathway analysis of differentially expressed miRNAs. KEGG pathway involvement was predicted using miRPathDB v.2.0 (https://mpd.bioinf.uni-sb.de/heatmap_calculator.html?organism=hsa; accessed September 25, 2024). Enrichment values are represented by a color gradient, with darker blue shades indicating higher levels of enrichment. **(A)** Heatmap of differentially expressed miRNAs in OSCC cells, highlighting strong associations of miR-142, miR-146a, and miR-223 with intracellular signaling cascades. **(B)** Heatmap of differentially expressed miRNAs in MCs, showing significant associations of miR-199a, miR-200b, miR-221, miR-200a, miR-34c, miR-494, and miR-125a with intracellular signaling pathways.

To investigate the functional implications of deregulated miRNAs, a KEGG pathway analysis was performed. This analysis integrates miRNA regulation with transcriptomic changes, providing a comprehensive view of molecular network dynamics. For OSCC cells, the analysis revealed significant enrichment of pathways related to cellular signaling, metabolism, and disease-associated mechanisms. Key pathways include apoptosis, cytokine-cytokine receptor interaction, chemokine signaling, TNF signaling, NF-κB signaling, Toll-like receptor signaling, FoxO signaling, NOD-like receptor signaling, PI3K/Akt signaling, cell cycle regulation, JAK/STAT signaling, p53 signaling, cytosolic DNA sensing, thyroid signaling, and Ras/Raf/MAPK signaling. These pathways were enriched with target genes regulated by miR-146a-3p, miR-146a-5p, miR-223-3p, miR-142-3p, and miR-142-5p. The heatmap (**Figure 6A**) highlights the expression levels of miRNAs associated with these pathways in OSCC cells, demonstrating distinct clustering of miRNAs targeting genes within the enriched KEGG pathways. For MCs, key enriched pathways included PI3K/Akt signaling, p53 signaling, Ras signaling, mTOR signaling, Ras/Raf/MAPK signaling, TNF signaling, chemokine signaling, cytokine-cytokine receptor interaction, RNA transport, JAK/STAT signaling, Toll-like receptor signaling, ERbB signaling, and FoxO signaling. These pathways were enriched with target genes primarily regulated by miR-199a-5p, miR-200b-3p, miR-221-3p, miR-200a-3p, miR-34c-5p, miR-494-3p, and miR-125a-5p. The heatmap (**Figure 6B**) illustrates distinct clusters of miRNAs targeting genes within these pathways, highlighting their regulatory roles. These findings underscore the critical involvement of miRNAs in regulating key molecular processes and their potential contributions to the interactions between MCs and OSCC.

### 3.6 Differential regulation of the CCL2/CCR2 axis and key signaling pathways in MC-OSCC interaction

**Figure 7:**
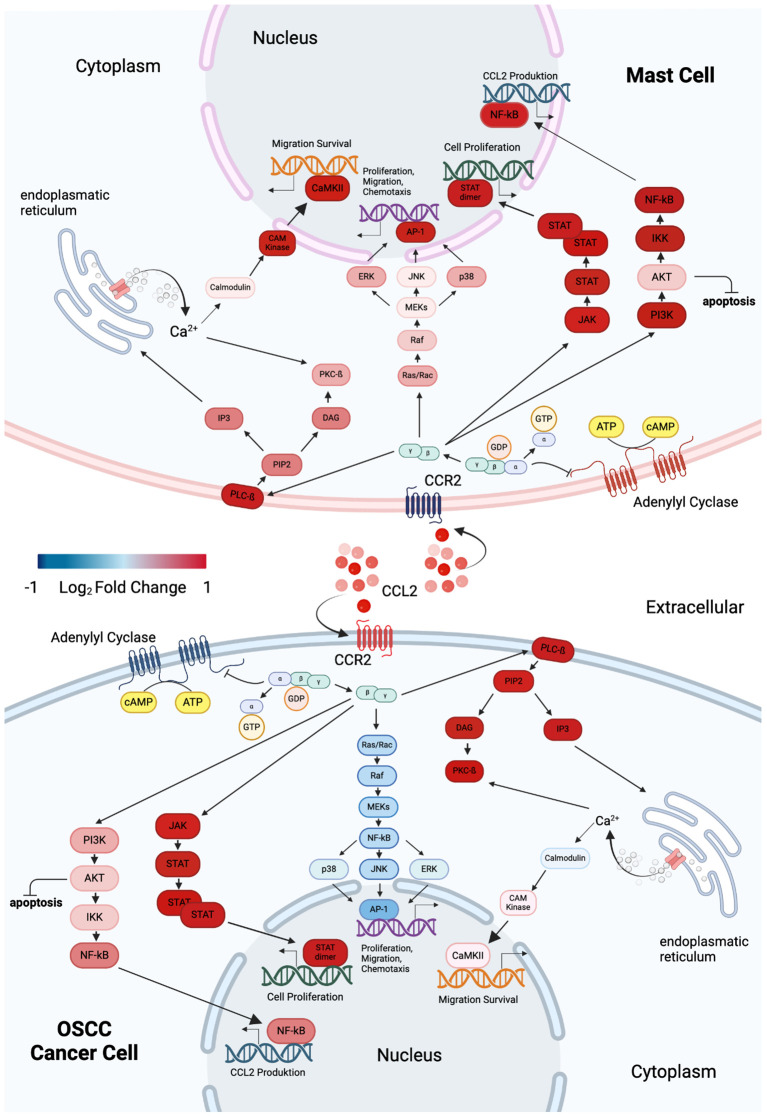
Graphical overview of DEGs in MCs and OSCC cells within CCL2/CCR2-associated pathways, highlighting changes in cellular properties. Blue indicates downregulation, while red signifies overexpression. The upper panel represents MCs, and the lower panel illustrates OSCC cells. Both cell types exhibit upregulation of CCL2 expression during co-culture. However, CCR2 expression shows opposing regulation: it is downregulated in MCs and upregulated in OSCC cells. Intracellular signaling in MCs activates the PI3K/Akt, JAK/STAT, Ras/Raf/MAPK, and IP3 pathways. In contrast, OSCC cells activate the PI3K/Akt, JAK/STAT, and IP3 pathways, with inhibition of the Ras/Raf/MAPK pathway. Graphic created with BioRender (www.biorender.com).

Transcriptomic analysis of MCs and OSCC cells after co-culture compared to monoculture controls revealed distinct regulatory patterns in genes associated with the CCL2/CCR2 axis and key signaling pathways (61) (**Figure 7**). In MCs, CCL2 expression was significantly upregulated while CCR2 expression was downregulated. This was accompanied by the upregulation of several genes in multiple pathways. Specifically, genes involved in the PI3K/Akt pathway (88) (PIK3R1, AKT2, IKBKB, and NFKB2) and the JAK/STAT pathway (89) (JAK3 and STAT2) were upregulated. Similarly, genes associated with the Ras/Raf/MAPK pathway (90) (RACGAP1, BRAF, MAPK9, MAPK14, MAPK3, and JUN) and the IP3 signaling pathway (91) (PLCB2, PIP5K1C, PRKCB, CALM3, and CAMK2B) showed increased expression. In contrast, OSCC cells showed upregulation of both CCL2 and CCR2 expression. PI3K/Akt pathway genes (88) (PIK3R1, AKT2, IKBKB, and NFKB2) and JAK/STAT pathway genes (89) (JAK3 and STAT2) were also upregulated. However, a different regulatory pattern was observed in the Ras/Raf/MAPK pathway (90), where the genes RACGAP1, BRAF, MAPK9, MAPK14, MAPK3, and JUN were downregulated. Furthermore, within the IP3 signaling pathway (91), PLCB2, PIP5K1C, PRKCB, and CAMK2B were upregulated, whereas CALM3 was downregulated. These findings highlight the differential regulation of the CCL2/CCR2 axis and related pathways in MCs and OSCC cells, reflecting their distinct biological roles and potential implications in disease mechanisms.

### 3.7 High CCR2 expression is significantly associated with improved overall survival in head and neck tumors

**Figure 8:**
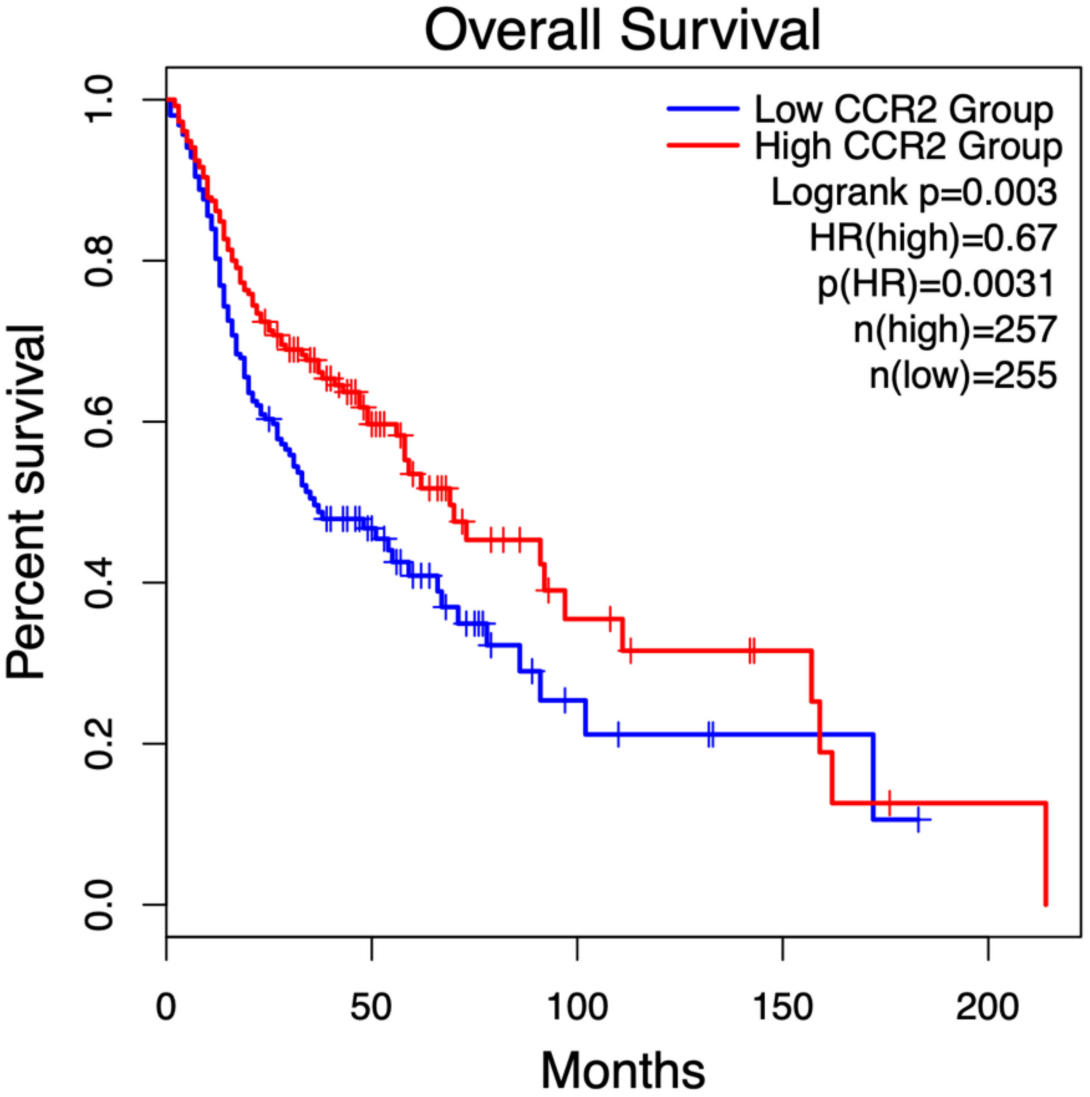
Kaplan-Meier survival analysis of CCR2 expression in head and neck tumor patients, stratified by median expression levels, was performed using the open GEPIA2 database (68) (gepia2.cancer-pku.cn; accessed January 2, 2025). Patients with high CCR2 expression (red curve, n = 257) exhibited a 33% lower risk of death compared to those with low CCR2 expression (blue curve, n = 255), as indicated by a hazard ratio (HR) of 0.67 (p = 0.0031). Statistical significance between survival curves was assessed using the log-rank test (p = 0.003).

The prognostic significance of identified key genes in OSCC was evaluated by analyzing their impact on overall survival (OS) in head and neck tumors using the open GEPIA2 database (68). Kaplan-Meier survival curves were generated and analyzed to assess the correlation between gene expression and survival outcomes. Among the key genes, only CCR2 expression showed a significant association, with high expression levels linked to improved OS, highlighting its potential prognostic value in head and neck tumors. The Kaplan-Meier plots (**Figure 8**) visually depict these findings.

### 3.8 Key miRNAs in OSCC are significantly associated with improved overall survival in head and neck tumors

**Figure 9:**
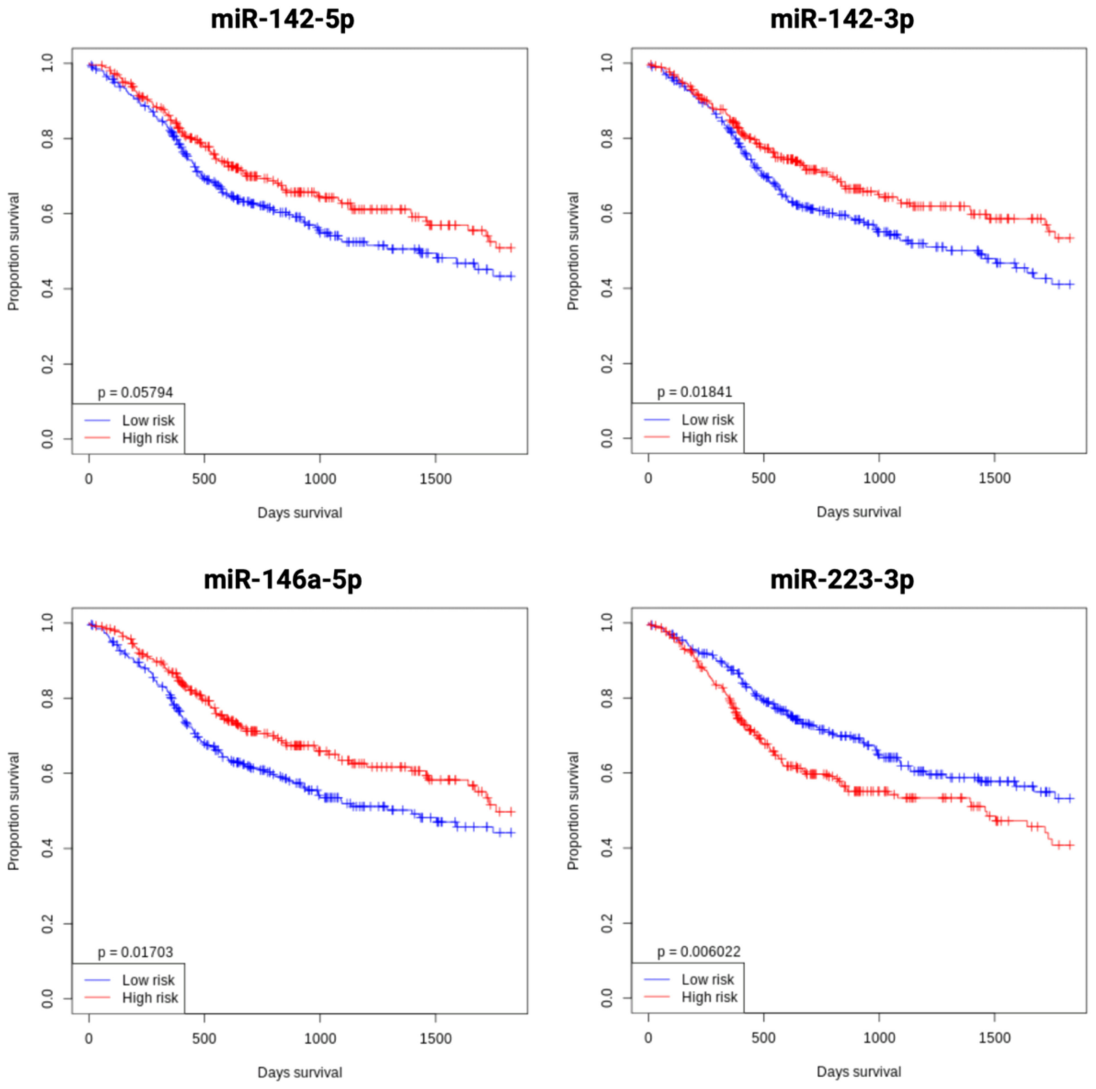
Kaplan-Meier overall survival analysis of key miRNAs (miR-142, miR-146a, and miR-223) in OSCC, stratified by median expression levels, using the open OncomiR database (oncomir.org; accessed January 2, 2025) (73). High expression (n = 261) is shown in red curves, and low expression (n = 261) in blue curves. Increased expression of miR-142-5p, miR-142-3p, and miR-146a-5p, as well as decreased miR-223-3p expression, correlate with improved overall survival in head and neck tumor patients. Statistical significance was assessed using the log-rank test.

The prognostic relevance of key miRNAs in OSCC was assessed by analyzing their impact on OS in head and neck tumor patients using the open OncomiR database (73). Kaplan-Meier survival curves were generated to evaluate the relationship between miRNA expression and patient survival. The analysis revealed significantly improved OS associated with the upregulation of miR-142-5p, miR-142-3p, and miR-146a-5p, as well as the downregulation of miR-223-3p. These findings underscore the potential of these miRNAs as prognostic biomarkers in head and neck tumors and as potential modulators of tumor progression. The Kaplan-Meier plots visually depict these associations (**Figure 9**).

## 4 Discussion

The role of MCs in tumor progression remains unclear as they exert both pro- and anti-tumorigenic effects depending on their localization within the TME or the tumor cell complex (30, 35, 36, 38–46). In OSCC, MCs predominantly infiltrate the TME, with only a small fraction detected within the tumor cell complex (58). However, data on their impact in OSCC remain scarce and conflicting (34, 92–94). Preliminary evidence suggests that high MC density in the TME correlates with improved overall survival, possibly due to their limited degranulation (58). The underlying cause of reduced degranulation remains unclear.

The mechanisms driving MC accumulation in the TME are also unknown, although tumor cells secrete chemokines that recruit immune cells, which may be exploited to support tumor progression (47). Conversely, MCs secrete chemokines that facilitate immune cell recruitment, potentially contributing to tumor defense (53, 95–97), suggesting a bidirectional interaction between MCs and OSCC.

Among the mediators of MC-OSCC interactions, CCL2 has been identified as a key chemokine associated with reduced tumor invasiveness (59). The transcriptome analysis in this study confirms this, revealing a significant upregulation of CCL2 and its receptor CCR2 in co-cultured OSCC cells, as validated by RT-qPCR. Clinically, high CCR2 expression correlates with prolonged overall survival in head and neck tumor patients. Notably, while CCL2 was significantly upregulated in MCs, CCR2 expression was downregulated, potentially serving as a countermeasure against pro-tumorigenic signaling. The intracellular signaling pathways modulated by MC-OSCC crosstalk via chemokines remain poorly understood. However, the CCL2/CCR2 axis is known to activate several intracellular pathways, including PI3K/Akt, JAK/STAT, Ras/Raf/MAPK, and IP3, which regulate key cellular functions such as proliferation, migration, and chemotaxis (61). Molecular analyses in this study suggest inhibition of the Ras/Raf/MAPK pathway in OSCC cells, which may explain the observed reduction in tumor invasiveness (59).

Other molecular mechanisms underlying MC-tumor interactions have been reported (47), some of which are consistent with the current findings in OSCC. The TGF-β signaling pathway appears to play a critical role, with a pronounced upregulation of TGF-β2 and a moderate increase in TGF-β1 expression in MCs. Gene set enrichment analysis (GSEA) confirmed differential expression of several TGF-β pathway-associated genes in MCs, whereas tumor cells showed minimal upregulation of TGF-β1/2. Toll-like receptors (TLRs), key regulators of immune responses, also showed distinct expression patterns in co-cultured MCs and OSCC cells. MCs showed significant upregulation of TLR3, TLR5, TLR6, and TLR8, whereas TLR2 was slightly downregulated. In contrast, OSCC cells showed downregulation of TLR5 and TLR6 with modest upregulation of TLR2 and TLR3. Notably, TLR2 activation has been associated with tumor cell proliferation and inhibition of apoptosis (98). Thus, the observed TLR2 upregulation in OSCC cells may result from MC interactions, contributing to their proliferative advantage, as suggested in preliminary studies (59). Furthermore, the increased expression of miR-146a in OSCC cells and miR-125a in MCs is consistent with their known regulatory roles in TLR signaling (99, 100).

Transcriptomic analysis further revealed differential gene expression in both OSCC cells and MCs. OSCC cells exhibited strong upregulation of COX-2 and mitochondrial genes (MT-ND2, MT-ND4, MT-ND5, and cytochrome b), indicating increased metabolic activity, a hallmark of tumor progression (98). Given the established role of COX-2 in inflammation and tumor progression, its increased expression may contribute to OSCC cell proliferation, invasion, and angiogenesis (98, 101, 102). In MCs, BTG2 emerged as a highly differentially expressed gene that plays a pivotal role in MC survival, migration, and cell cycle regulation. Its overexpression suppresses MC proliferation and mediator release (103), which may explain the limited degranulation observed in OSCC (58). In addition, BTG2 modulates transcription factors involved in tumor proliferation (104), suggesting that its high expression in MCs may represent an OSCC-driven mechanism to evade immune responses. Conversely, the upregulation of SKIL, which promotes MC proliferation and cytokine production (105–107), suggests a potential anti-tumor effect in the TME. The differential expression of EXOC6B, a key component of MC degranulation, along with SERPINE1 and BHLHE40, further highlights the intricate regulation of MC function within the OSCC TME (106, 108–110). The downregulation of MLC1, a regulator of mediator exocytosis, suggests reduced histamine release, which may contribute to the observed limited MC degranulation (58).

Distinct miRNA expression profiles have been identified as potential regulators of MC-OSCC interactions. In tumor cells, elevated levels of miR-142 and miR-146a correlated with a favorable prognosis in patients with head and neck tumor. miR-142 suppresses carcinogenesis by inhibiting tumor proliferation and invasion (111–113), whereas miR-146a modulates NF-κB signaling to impede tumor progression (114, 115), a finding supported by survival analysis in this study. In contrast, high miR-223 expression was associated with poor prognosis, consistent with its dual role in cancer progression and drug resistance (116). In MCs, miR-381 and miR-379 have been identified as regulators of inflammatory and cytokine pathways (117, 118). Overexpression of miR-379, which inhibits tumor cell invasion and EMT (119, 120), suggests a potential role in attenuating OSCC progression.

This study uncovers a complex regulatory network governing MC-OSCC interactions, characterized by significant transcriptomic and miRNA alterations. Key findings include the involvement of the CCL2/CCR2 axis, inhibition of the Ras/Raf/MAPK pathway in OSCC, and the prognostic significance of miR-142, miR-146a, and miR-223. These molecular signatures may serve as promising therapeutic targets for OSCC intervention.

## 5 Conclusions

The interaction between MCs and OSCC significantly alters the transcriptome and miRNA expression profiles in both cell types, indicating a complex intercellular regulatory network. In particular, the CCL2/CCR2 axis, the inhibition of the Ras/Raf/MAPK pathway, and the differential expression of miR-142, miR-146a, and miR-223 in tumor cells appear to play a critical role and have high clinical relevance. However, the study’s reliance on an *in vitro* co-culture model and single cell lines limits its ability to fully capture the complexity of the TME. Future research should focus on validating the role of CCL2 signaling and the broader molecular mechanisms of MC-OSCC interactions *in vivo* to assess their functional relevance and therapeutic potential.

## Data Availability

The datasets presented in this study can be found in online repositories. The names of the repository/repositories and accession number(s) can be found below: https://www.ncbi.nlm.nih.gov/geo/, GSE278247 https://www.ncbi.nlm.nih.gov/geo/, GSE278404.
